# Flame Retardancy Properties and Physicochemical Characteristics of Polyurea-Based Coatings Containing Flame Retardants Based on Aluminum Hydroxide, Resorcinol Bis(Diphenyl Phosphate), and Tris Chloropropyl Phosphate

**DOI:** 10.3390/ma14185168

**Published:** 2021-09-09

**Authors:** Wojciech Dukarski, Piotr Krzyżanowski, Marcin Gonsior, Iwona Rykowska

**Affiliations:** 1 Faculty of Chemistry, Adam Mickiewicz University, Uniwersytetu Poznańskiego 8, 61-614 Poznań, Poland; obstiwo@amu.edu.pl; 2 STI Chemsampler Sp. z o. o. Sp. k., Romana Maya 1, 61-371 Poznań, Poland; piotr.krzyzanowski@sti-izolacje.pl (P.K.); marcin.gonsior@sti-izolacje.pl (M.G.)

**Keywords:** polyurea, flame retardancy, thermal stability, thermogravimetric analysis, oxygen index, tensile strength, infrared spectroscopy, scanning electron microscopy

## Abstract

Polyurea is a synthetic material made by the reaction of isocyanate and polymer blend-containing amines. Due to its outstanding mechanical properties and fast curing, polyurea-based coatings have found dozens of applications, including waterproofing and anti-corrosion coatings. Further development of this material can create a flame-retardant product, a good alternative for common products available on the market, such as intumescent coatings. To improve the flame retardancy of polyurea, several flame retardants were investigated. The influence of aluminum hydroxide, resorcinol bis(diphenyl phosphate) (RDP), and tris chloropropyl phosphate (TCPP) on flame retardancy and morphology was studied. The following methods were used: infrared spectroscopy, scanning electron microscopy, thermogravimetric analysis, limiting oxygen index, and tensile strength. The examinations mentioned above showed the improvement of flame-retardancy of polyurea for two products: chlorinated organophosphate and organophosphate. Nevertheless, using the chlorinated organophosphate additive caused a rapid deterioration of mechanical properties.

## 1. Introduction

Fire resistance is one of the most challenging issues raised in the building design process. Building elements such as roofs or steel constructions are in the greatest danger when exposed to fire. The structure of the roofs is typically based on wood. Time to ignition for such elements may be extremely short, often less than 1 min [1]. Hence, they lose their mechanical parameters rapidly. Roofs based on steel constructions also do not remain indifferent in the event of a fire. Steel, being generally a fire-resistant material, may become a great danger when the fire is fully developed. This problem is often described in the literature [2,3,4]. At a temperature over 600 °C, steel loses two-thirds of its yield strength. Hence, it can no longer perform a construction function and may cause a collapse of an entire building. Nowadays, steel is used in almost every field of construction, including buildings, ships, and automobiles. To improve fire retardancy, protecting layers, such as intumescent coatings or mineral wood, need to be applied. Polyurea-based coatings with increased flame retardancy may be a beneficial alternative for such products. Polyurea and polyurethane–polyurea hybrids are usually used as a podium deck and foundation wall waterproofing. However, there are many more applications, such as roof coatings, pipe protection, secondary containers, bridge deck waterproofing, tank liners, automotive industry, pool coatings, and many more [5,6,7]. Thanks to continuous material development, the number of applications is growing. Polyurea shows excellent properties, including fast curing (even at low temperatures), water resistance, insensitivity to air moisture, and comprehensive chemical resistance [8,9]. This material shows excellent mechanical properties, including tensile strength (usually greater than 20 MPa), high elongation at break (up to 500%), and excellent adhesion to a variety of surfaces [10,11]. Nevertheless, polyurea coatings are assigned to the European fire class E, according to EN 1350-1 [12]. Hence, this material is poorly resistant to fire exposure. A general chemical structure of the polyurea molecule shows that this compound can sustain the burning process. It is caused by its organic nature and the presence of oxygen in its chain. Higher temperatures (above 250–300 °C) cause the decomposition of most chemical bonds in the chemical structure and make the burning process challenging to stop [13]. With improved resistance to fire, polyurea can extend its use to some applications where fireproofing properties are needed, e.g., roof coatings, blast mitigation, ballistic protection flooring in explosion hazard zones, or even steel construction fireproofing.

There are a variety of flame retardants available on the market [14]. Mainly, they are classified into two groups, including additive and reactive compounds. Additive flame retardants are inert to the polymer matrix, usually acting as a cooling substance when exposed to the fire. They include primarily hydroxides (aluminum, magnesium), but also compounds containing antimony, phosphorus, chlorine, and bromine elements. In general, the additive compounds are cheaper, but it is often required to use a relatively large amount to obtain a material with sufficient inflammability properties (up to 60%), e.g., aluminum trioxide. Reactive flame retardants are prepared with resin, creating an integrated part of the polymeric network. For the most part, they are made of bromine, chlorine, phosphorus, melamine, and inorganic compounds. Halogen compounds are exceptionally desired due to their efficiency in inhibiting flames [15]. Besides these, a rather newly developed group, intumescent flame retardants (IFR), are available on the market. They are made of acid, carbon, and gas sources, and their behavior is based on creating a swollen layer under the fire exposition. However, IFR exists as a thin protective coating that must be applied on a flammable surface in the form of a paint [16,17].

In this study, flame retardants based on aluminum hydroxide, RDP, and TCPP were examined, and their influence on physicochemical and flammability properties was determined. The effect of the chosen additives was defined using several laboratory methods, including Fourier transform infrared spectroscopy, scanning electron microscopy, thermogravimetric analysis, limiting oxygen index, and tensile strength.

## 2. Materials and Methods

### 2.1. Samples’ Preparation

The materials used throughout this work are the following: low-functionality MDI-based prepolymer containing 15% of NCO groups (Huntsman, Texas, CA, USA), diamine (Albemarle, Charlotte, NC, USA), a chain extender (Lonza, Basel, Switzerland), polyether polyol (PCC Rokita, Brzeg Dolny, Poland), aliphatic diol (LyondellBasell, Rotterdam, Netherlands), aluminum hydroxide, resorcinol bis(diphenyl phosphate), and tris chloropropyl phosphate. Table 1 presents the flame retardants used and their abbreviations.

The samples were prepared in a few steps. In the beginning, the polyol blend (Part B) was made of diamine (10–15% pbw), a chain extender (10–15% pbw), polyether polyol (30–60% pbw), aliphatic diol (2–10% pbw), flame retardant (10–30%), and a necessary catalyst by mixing them in a beaker at a calculated ratio, and a homogenous mixture was obtained by using a high-speed stirrer. Part B prepared in this way was poured to cartridge together with unmodified isocyanate (part A) and sprayed onto polypropylene (PP) boards in the volume ratio of 1:1. In the formula calculations, a 1.1 isocyanate index was established. The polyurethane–polyurea hybrid samples were cured at room temperature for seven days. The amount of FR was set at 5%, 10%, and 15% by the total weight of the compound. A reference sample without FR was also prepared following the above procedure.

### 2.2. Methods

#### 2.2.1. Fourier Transform Infrared Spectroscopy (FTIR) 

The FTIR spectra of polyurea–polyurethane copolymers were examined using a Bruker IFS 113 V spectrophotometer with the single quest reflection ATC accessory 187 IR affinity-1. Samples were tested in the 400–4000 cm^−1^ range at a resolution of 2 cm^−1^. Five samples in the series were established. All characteristic wavenumbers were registered and compared with the reference sample.

#### 2.2.2. Scanning Electron Microscopy (SEM)

The analysis of the surface of sprayed coatings was performed on a scanning electron microscope Hitachi SU3500. Each series consisted of five trials. This technique was used for investigating the flame retardants’ influence on the surface appearance. Every imperfection, such as scratches, pores, and microfractures, was interpreted. A 5000 times magnification was defined.

#### 2.2.3. Thermogravimetric Analysis (TGA)

According to standard method PN-EN ISO 11,358 [18], mass loss was investigated with the Netzsch TG 209 Libra apparatus. Small specimens (approximately 10 mg) were prepared. The scanning was performed at temperatures ranging from 20 to 1000 °C at a heating rate of 5 °C/min in the air atmosphere. Each series was performed five times. The thermal degradation curve and a final residue were determined for each sample.

#### 2.2.4. Limiting Oxygen Index (LOI)

**Figure 1:**
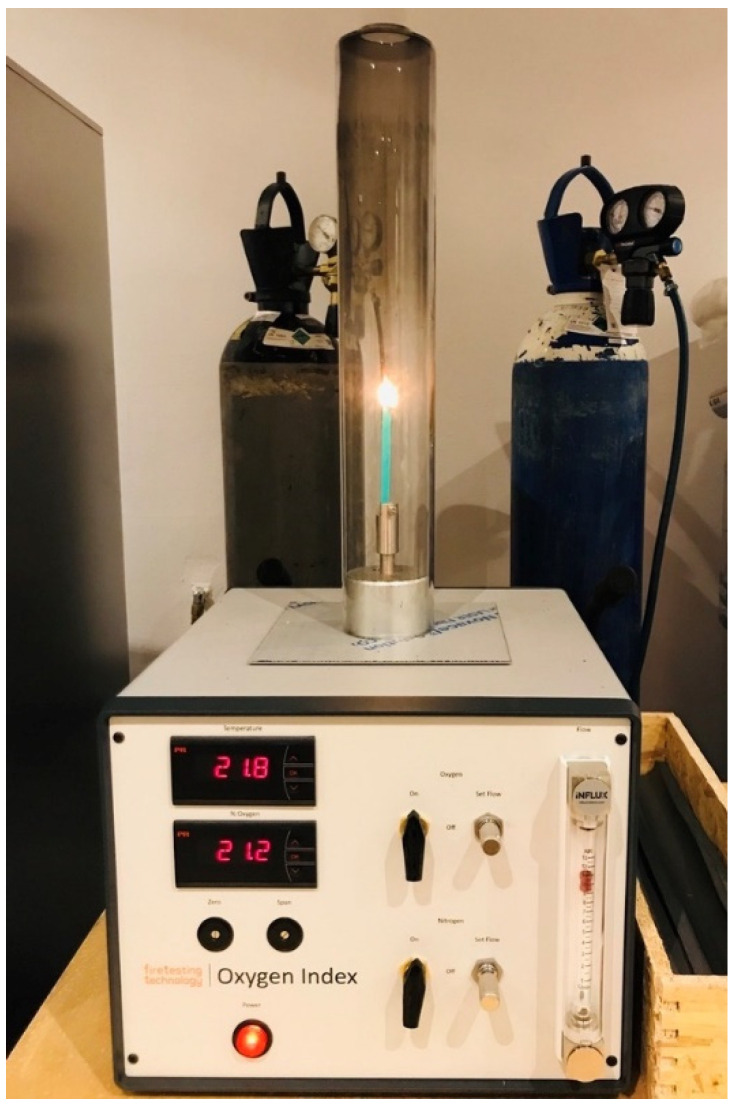
Fire-Testing Technology apparatus.

According to standard method ISO 4589-2 [19], LOI was investigated using a Fire-Testing Technology apparatus (Figure 1). Samples for testing had 140 cm length and 52 cm width. All samples had an average thickness of 2 mm. The minimum concentration of oxygen in the oxygen–nitrogen mixture required to sustain the combustion process was determined. Ignition procedure A with at least eight burning attempts was chosen.

#### 2.2.5. Tensile Tests

The mechanical durability was investigated on a 10 kN INSTRON 34TM-30 tensile testing machine in accordance with standard method ISO 527-2: “Plastics—Determination of tensile properties” [20]. Type 1BA of the specimen’s shape was selected. Each series consisted of five trials. Stress–strain curves were drawn. Maximum stress and elongation at break were determined and compared. Both the elongation at break and maximum stress can be effectively used to estimate the compatibility between the flame retardant and the polymeric matrix.

## 3. Results

This section describes the influence of flame retardants on the chemical, physical, and thermal properties of polyurea-based coatings compared with a standard recipe without FR content. 

### 3.1. Chemical Composition

**Figure 2:**
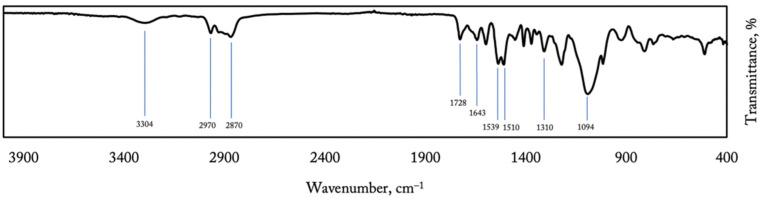
FTIR spectrum taken on reference sample.

Figure 2 presents a spectrum of unmodified polyurea–polyurethane composite. Several characteristic bands are visible: a very strong absorption peak at 1094 cm^−1^, attributed to the CN stretch alkyl amine group, strong peaks at 1510 and 1539 cm^−1^ connected with NO_2_ bonds, peaks at 1643 and 1728 cm^−1^ indicating urea and urethane groups, and CH stretch alkane and aldehyde groups at 2970 and 2870 cm^−1^, respectively. Furthermore, a stretch carboxylic absorption peak at 1310 cm^−1^ and an amide bond at 3304 cm^−1^ were indicated [21,22,23].

**Figure 3:**
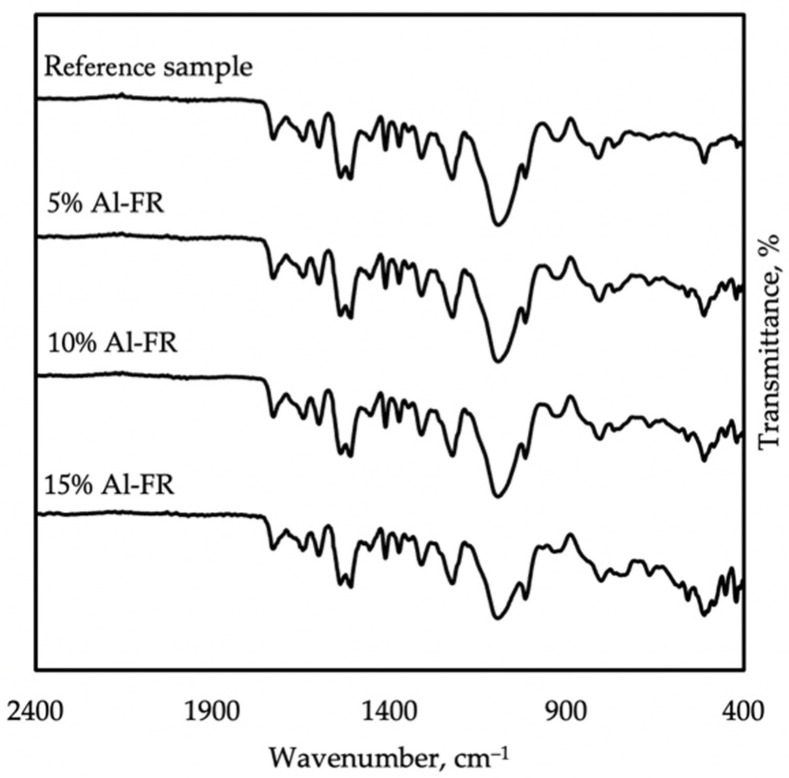
FTIR spectra taken on reference samples, 5% Al-FR, 10% Al-FR, and 15% Al-FR, showing the influence of aluminum hydroxide.

Formulations of polyurea–polyurethane coatings modified with flame retardants were also examined with FTIR spectroscopy. The addition of aluminum hydroxide showed no new bands in the spectrum (Figure 3). Most of the absorption peaks decreased, caused by a more significant content of inorganic compounds in the membrane composition. The fingerprint region between 400 and 800 cm^−1^ was intensified. They are connected with bending vibrations within the molecule. The fingerprint region is mainly used as a confirmation of a molecule’s identity [23,24].

**Figure 4:**
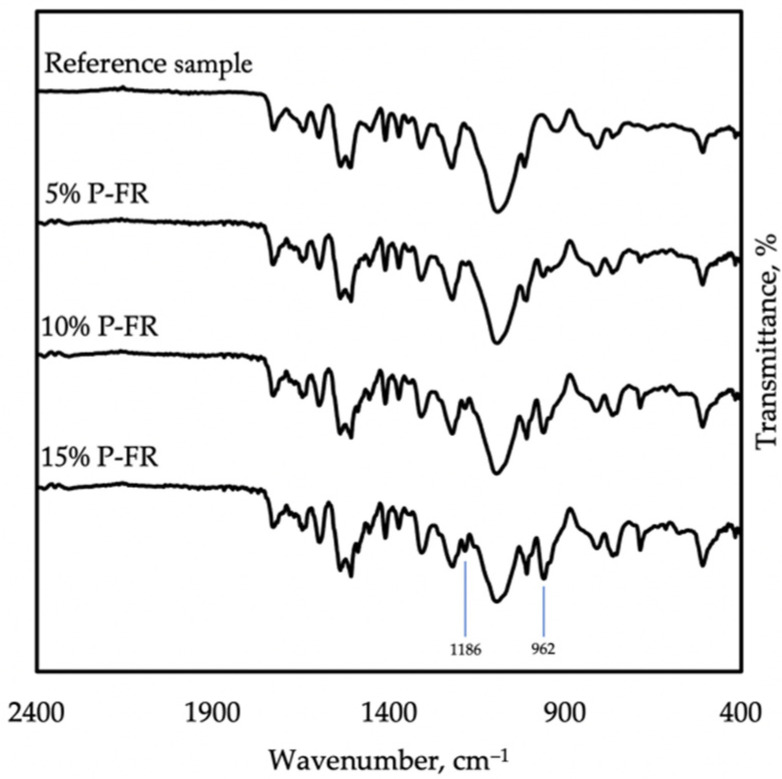
FTIR spectra taken on reference samples, 5% P-FR, 10% P-FR, and 15% P-FR, showing the influence of RDP.

The addition of resorcinol bis(diphenyl phosphate), as shown in Figure 4, formed new bands: at 962 cm^−1^ attributed to PO single bond, and at 1186 cm^−1^ related to the P-O-Ar group. Furthermore, the fingerprint region has been changed [22].

**Figure 5:**
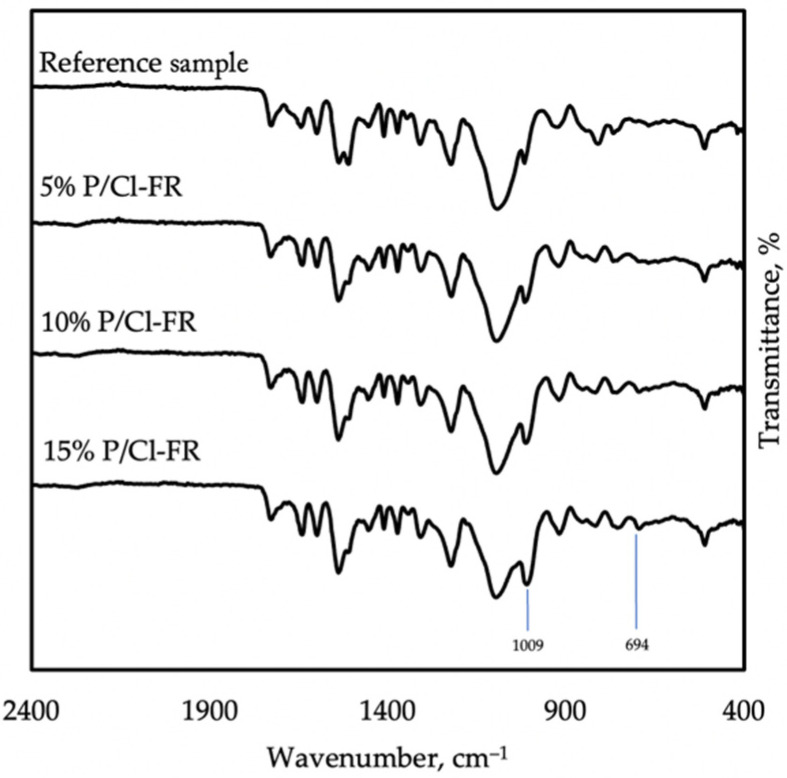
FTIR spectra taken on reference samples, 5% P/Cl-FR, 10% P/Cl-FR, and 15% P/Cl-FR, showing the influence of TCPP.

The presence of tris chloropropyl phosphate in the chemical structure of polyurea was registered in the spectrum (Figure 5). The strongest peak coming from this compound was seen at 1009 cm^−1^, indicating the P-O-C group. Furthermore, a C-Cl bond was visible at 694 cm^−1^ [22].

### 3.2. Surface Analysis

**Figure 6:**
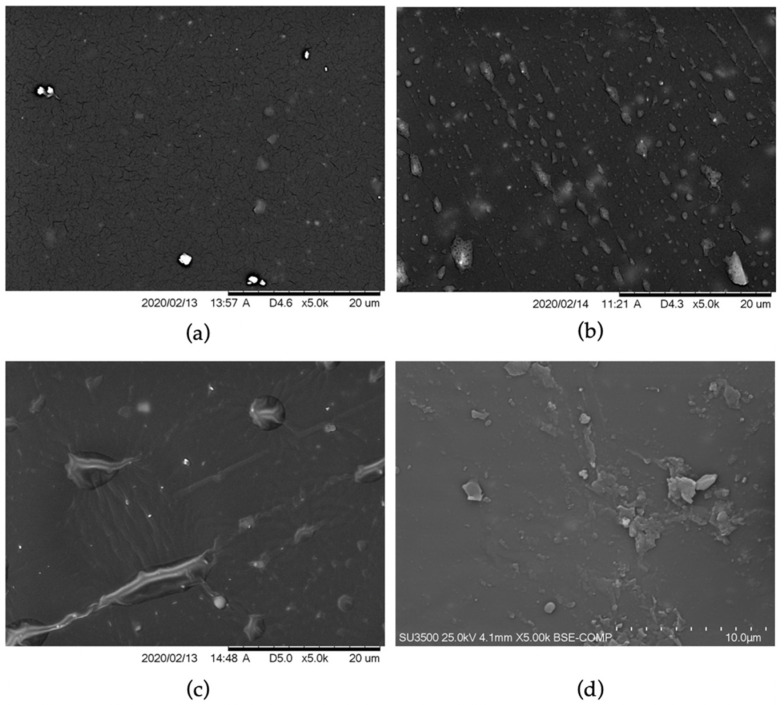
SEM of elastomeric hybrids based on polyurea: (**a**) reference sample; (**b**) 10% Al-FR; (**c**) 10% P-FR; (**d**) 10% P/Cl-FR.

The addition of flame retardants can cause imperfections on the surface, making the material less durable. SEM images of a reference sample were compared with images of samples at 10% loading of an FR (Figure 6). The surface of the unmodified sample showed a large number of microcracks. In the other pictures, related to modified formulations, different phases are visible. They can be caused by an improper mixing ratio during spraying, flame retardants’ agglomeration, or curing conditions. The literature shows that microcracks can be developed from micropores created by violet radiation [25]. The significant impact of the recorded cracks was not observed during tensile tests.

### 3.3. Thermal Decomposition

**Figure 7:**
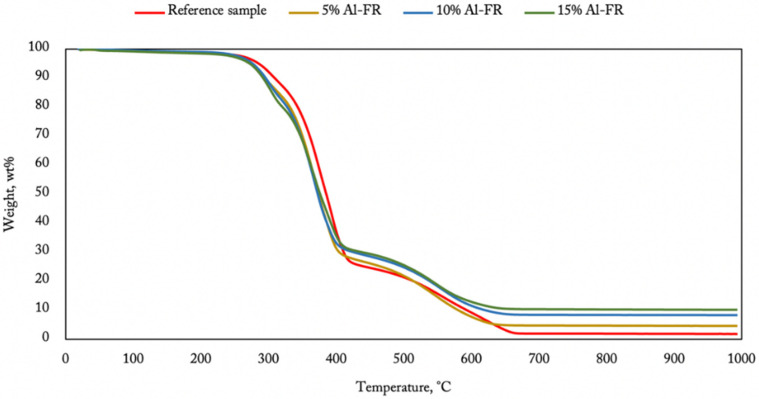
TGA of polyurea–polyurethane coatings containing aluminum hydroxide.

**Figure 8:**
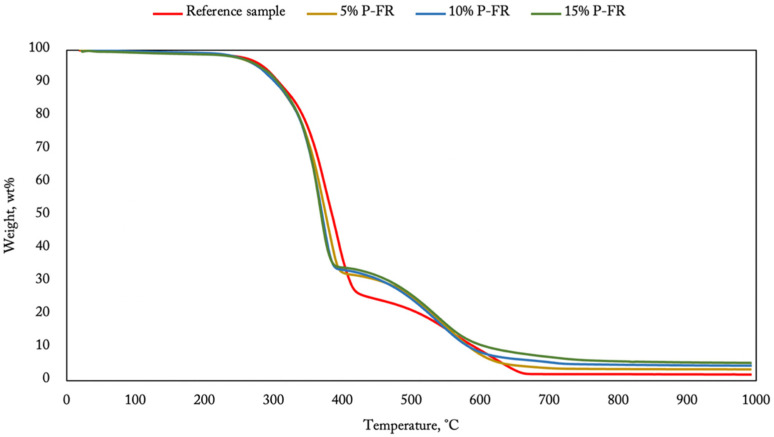
TGA of polyurea–polyurethane coatings containing RDP.

**Figure 9:**
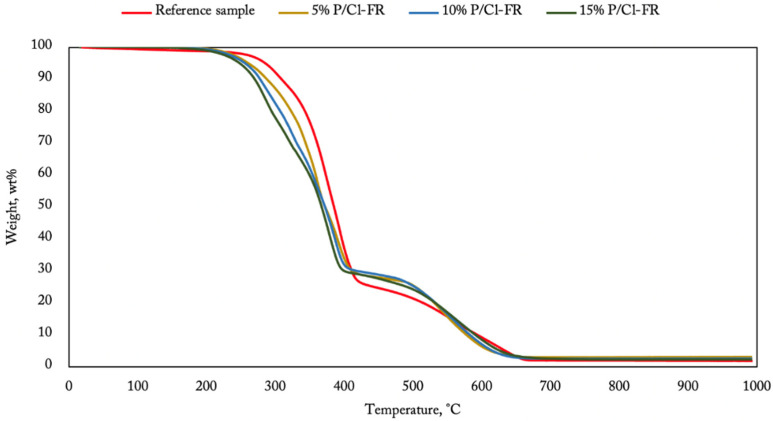
TGA of polyurea–polyurethane coatings containing TCPP.

Figure 7, Figure 8 and Figure 9 show the TGA results obtained for membranes containing flame retardants in the air atmosphere. It is easy to observe (Figure 7) how the inorganic compound content changes the terminal mass of the burnt sample. It is worth noticing that the decomposition pace was faster in the first phase for the 15% content of aluminum hydroxide. This phenomenon relates to water release from the aluminum compound that starts at 250 °C, going on to lose around 35% of its initial mass at 600 °C [26]. Water performs a cooling function and cannot be registered using thermogravimetry analysis. Flame retardancy improvement of the polyurea coatings containing aluminum hydroxide appeared when the temperature over 400 °C was reached.

A similar phenomenon can be observed for additives based on resorcinol bis(diphenyl phosphate) (Figure 8). The degradation process was faster for the modified coating until the temperature of 400 °C was reached. Over this temperature, the process slows down, providing the best efficiency between 400 and 550 °C. The terminal mass increased with a higher RDP content in the composition. The flame retardant composed of TCPP (Figure 9) provided a different result than the other additives. The degradation process started early at 200 °C, and it is the moment connected with the degradation and volatilization of the flame retardant. During this process, chlorine radicals are created to scavenge free OH• and H• radicals, indicating a flame [27,28,29].

### 3.4. Limiting Oxygen Index

Results for the limiting oxygen index analysis are presented in Table 2. The oxygen index determined for the reference sample shows that 21.6% ± 0.163% of oxygen content is enough to sustain the flame. This is a comparable value to an oxygen content we can find in the air. Due to this result, it is hard to predict if this material burns under normal conditions. Black, dense smoke was observed during the burning process. This phenomenon is related to incomplete combustion caused by the presence of aromatic rings in the chemical structure [30]. The addition of aluminum hydroxide surprisingly decreased the LOI value by over 2% for the 15% aluminum compound content (19.3% ± 0.151%). This result is not consistent with the literature results [31,32]. It shows its poor flammability properties for polyurea/polyurethane applications. The sample containing the aluminum compound was burning faster than the reference sample, and dripping appeared during testing. A much better result was obtained from RDP and TCPP, increasing LOI levels up to 22.8% and 24.1%, respectively. Both additives formed a protective char layer, reducing the amounts of combustible gases. This phenomenon was caused by the presence of phosphorus in the chemical structure [27]. Chlorine, present in the molecule of tris chloropropyl phosphate, effectively inhibited the combustion process in the gas phase by quenching free radicals.

### 3.5. Tensile Strength

**Figure 10:**
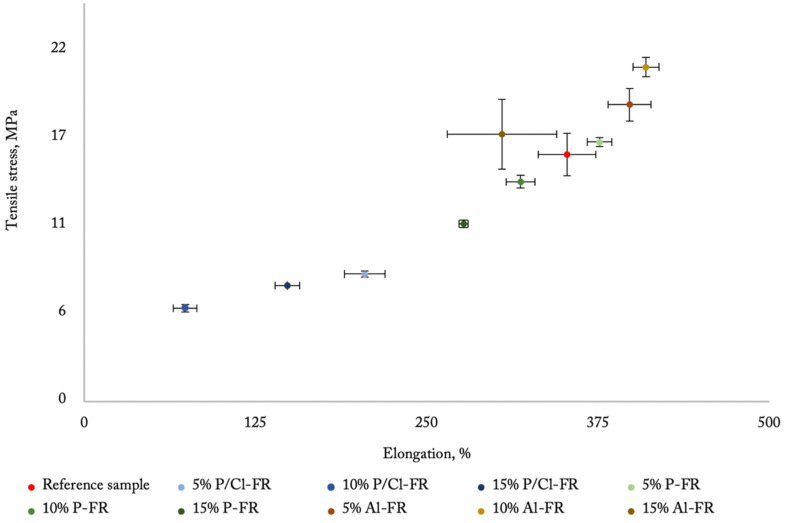
Tensile test of polyurea-based hybrids.

The tensile strength results are presented in Figure 10. The tensile stress obtained 15.5 MPa, and the elongation at break value reached over 352% for the reference sample. Modified polyurea formulations showed a wide range of mechanical strength. The best result was obtained with aluminum hydroxide. The content of 10% of this flame retardant caused the increase of maximum tensile stress, up to 21.0 MPa. The elongation at break was also improved, reaching almost 410%. This effect is caused by the reinforcing nature of mineral additives. The 15% content of the aluminum-based flame retardant showed mechanical properties close to the reference sample. The worst durability result was obtained with the TCPP flame retardant, where the 5% content of this additive caused a decrease of maximum tensile stress from 15.5 to 8 MPa, and elongation at break from 352% to 205%. The negative effect of this additive on mechanical properties was not investigated with polyurea or polyurethane compounds. The addition of the RDP flame retardant had the most negligible influence on the mechanical properties of modified coatings, where the 5% content of this additive caused the improvement of elongation at break and maximum tensile stress, which were 376% and 16.3 MPa, respectively.

## 4. Discussion

Tests results shown in this work proved the difficulty of improving inflammability of polyurea-based coatings. Quite often, obtaining better flame retardancy causes the deterioration of mechanical durability. For mineral additives, a content of 50% or more is needed to obtain satisfying inflammability of a material [33]. For this reason, this type of flame retardant is not recommended. Furthermore, due to difficult manufacturing conditions (high pressure > 150 bar [8]), the flame retardants should have limited viscosity, often not higher than 1000 mPa·s. Within this work, the flame retardancy improvement of polyurea-based coatings containing organophosphorus compounds was proven. However, it is essential to carry out additional tests verifying the behavior of modified polyurea-based coatings in real applications. Further studies will also include cone calorimetry examination to describe the combustion process and determine properties such as the heat release rate, the mass-loss rate, the gas generation, or the ignition time. The UL-94 test will also be performed. The effect of flame retardants’ compositions on flammability will be investigated.

## 5. Conclusions

In this work, the influence of aluminum hydroxide, resorcinol bis(diphenyl phosphate), and tris chloropropyl phosphate on the flammability of polyurea-based hybrids was determined. 

The addition of aluminum hydroxide showed no changes in the FTIR spectrum and did not show surface damage during SEM examination. Preliminary studies of TG showed an improvement in fire resistance. Nevertheless, the LOI test showed a poor influence of this flame retardant on the flammability properties of polyurea-based elastomers. The aluminum-based additive performed its suitable reinforcing property during the strength test, increasing the tensile stress up to 21 MPa. 

The presence of RDP flame retardant was proven by FTIR spectroscopy. Some imperfections of the surface were recorded by scanning electron spectroscopy, but no influence on mechanical properties was noticed. The flame retardant based on resorcinol bis(diphenyl phosphate) improved the flammability properties of polyurea-based elastomers, increasing the LOI by up to 22.8%. At the same time, mechanical properties were not deteriorated. Additionally, during the combustion process, char formation was observed.

The best flammability impact was found for a tris chloropropyl phosphate additive. The effect is visible in the LOI results, providing increase to 24.1%. Its good behavior was also proven by TG analysis, showing early decomposing of chlorine bonds at 200 °C. Unfortunately, the good flammability properties caused the deterioration of membrane durability.

The tests performed showed that the best effect was obtained with the flame retardant based on RDP. The low content (around 10%) of this additive effectively increased both oxygen index and mechanical properties.

## Figures and Tables

**Table 1:** Flame retardants.

Flame Retardant Name	Abbreviation
Aluminum hydroxide	Al-FR
Resorcinol bis(diphenyl phosphate)	P-FR
Tris chloropropyl phosphate	P/Cl-FR

**Table 2:** Limiting oxygen index analysis.

Sample Name	OI (%)
Reference sample	21.6
5% Al-FR	20.3
10% Al-FR	20.0
15% Al-FR	19.3
5% P-FR	21.6
10% P-FR	22.8
15% P-FR	22.3
5% P/Cl-FR	22.1
10% P/Cl-FR	23.1
15% P/Cl-FR	24.1

## Data Availability

Not applicable.
